# Correlation Between Fe/S/As Speciation Transformation and Depth Distribution of *Acidithiobacillus ferrooxidans* and *Acidiphilium acidophilum* in Simulated Acidic Water Column

**DOI:** 10.3389/fmicb.2021.819804

**Published:** 2022-02-09

**Authors:** Yu-hang Zhou, Can Wang, Hong-chang Liu, Zhen Xue, Zhen-yuan Nie, Yue Liu, Jiao-li Wan, Yu Yang, Wen-sheng Shu, Jin-lan Xia

**Affiliations:** ^1^Key Lab of Biometallurgy of Ministry of Education of China, School of Minerals Processing and Bioengineering, Central South University, Changsha, China; ^2^School of Life Sciences, South China Normal University, Guangzhou, China

**Keywords:** Fe/S/As speciation transformation, Fe/S redox reactions, *Acidithiobacillus ferrooxidans*, *Acidiphilium acidophilum*, acid mine drainage

## Abstract

It is well known that speciation transformations of As(III) vs. As(V) in acid mine drainage (AMD) are mainly driven by microbially mediated redox reactions of Fe and S. However, these processes are rarely investigated. In this study, columns containing mine water were inoculated with two typical acidophilic Fe/S-oxidizing/reducing bacteria [the chemoautotrophic *Acidithiobacillus (At.) ferrooxidans* and the heterotrophic *Acidiphilium (Aph.) acidophilum*], and three typical energy substrates (Fe^2+^, S^0^, and glucose) and two concentrations of As(III) (2.0 and 4.5 mM) were added. The correlation between Fe/S/As speciation transformation and bacterial depth distribution at three different depths, i.e., 15, 55, and 105 cm from the top of the columns, was comparatively investigated. The results show that the cell growth at the top and in the middle of the columns was much more significantly inhibited by the additions of As(III) than at the bottom, where the cell growth was promoted even on days 24–44. *At. ferrooxidans* dominated over *Aph. acidophilum* in most samples collected from the three depths, but the elevated proportions of *Aph. acidophilum* were observed in the top and bottom column samples when 4.5 mM As(III) was added. Fe^2+^ bio-oxidation and Fe^3+^ reduction coupled to As(III) oxidation occurred for all three column depths. At the column top surfaces, jarosites were formed, and the addition of As(III) could lead to the formation of the amorphous FeAsO_4_⋅2H_2_O. Furthermore, the higher As(III) concentration could inhibit Fe^2+^ bio-oxidation and the formation of FeAsO_4_⋅2H_2_O and jarosites. S oxidation coupled to Fe^3+^ reduction occurred at the bottom of the columns, with the formations of FeAsO_4_⋅2H_2_O precipitate and S intermediates. The formed FeAsO_4_⋅2H_2_O and jarosites at the top and bottom of the columns could adsorb to and coprecipitate with As(III) and As(V), resulting in the transfer of As from solution to solid phases, thus further affecting As speciation transformation. The distribution difference of Fe/S energy substrates could apparently affect Fe/S/As speciation transformation and bacterial depth distribution between the top and bottom of the water columns. These findings are valuable for elucidating As fate and toxicity mediated by microbially driven Fe/S redox in AMD environments.

## Introduction

Arsenic (As) pollution in acidic environments has been a global concern due to its harmfulness to the ecosystem and human health. The toxicity of As is determined by its speciation and occurrence forms that usually change with the variation of physicochemical properties of the surrounding environments (Cheng et al., 2009; Herath et al., 2016). Compared with arsenate [As(V)], arsenite [As(III)] is more difficult to remove from water and is generally considered to be more toxic (Kochhar et al., 1996). Microbially mediated dissolution of As-containing sulfide minerals, such as arsenopyrite (AsFeS), löllingite (FeAs_2_), and skutterudite [(Co, Ni)As_3–_*_*x*_*] (Corkhill and Vaughan, 2009; Drahota and Filippi, 2009; Johnson et al., 2020), is the main source for As contamination, in which microbially mediated iron/sulfur (Fe/S) redox reactions drive As speciation transformation to As(III) vs. As(V) as well as As release and thus As toxicity (Smedley and Kinniburgh, 2002; Burton et al., 2013; Wang et al., 2020).

On the one hand, during the oxidative dissolution of As-containing sulfide minerals driven by microbial Fe/S oxidation in acidic environments such as acid mine drainage (AMD) sites, a series of iron/sulfur-containing intermediates and secondary products are formed, e.g., orpiment (As_2_S_3_), jarosite (M[Fe_3_(OH)_6_(SO_4_)_2_], M = K^+^, Na^+^, NH_4_^+^, or H_3_O^+^), scorodite (FeAsO_4_⋅2H_2_O), and schwertmannite [Fe_8_O_8_(OH)_8–2x_(SO_4_)_x_ (where 1 ≤ *x* ≤ 1.75)], leading to changes in As speciation and occurrence forms due to As adsorption to or coprecipitation with the Fe/S secondary products (Zhang et al., 2019; Tabelin et al., 2020; Battistel et al., 2021). On the other hand, several acidophilic bacteria and archaea in acidic environments as well as neutrophilic Fe(III)- and sulfate-reducing bacteria (IRB and SRB, e.g., *Shewanella* and *Desulfovibrio*, respectively) in pH-neutral and mildly alkaline environments can lead to the reductive dissolution of Fe(III) minerals with the formation of Fe/S secondary products, such as mackinawite (FeS) and siderite (FeCO_3_), with a release of As species and the reduction of As(V) to As(III) (Drahota et al., 2013; Wang et al., 2016; Hedrich and Schippers, 2021).

It is well known that in acidic AMD environments, the gradients of pH, redox potential (ORP), dissolved oxygen (DO), and ion concentrations can significantly affect the microbial community and Fe/S redox activities (Chen et al., 2016; Han et al., 2018; Liu et al., 2019). For instance, in mill tailings, which can present a heterogeneous environment with extreme physicochemical properties, pH is primarily responsible for shaping the whole microbial community structure (Korehi et al., 2014; Liu et al., 2014). Besides, the secondary products formed due to microbial Fe/S redox cycling in AMD environments vary considerably at different pH values; e.g., schwertmannite is formed at pH 3–4.5, but jarosite is more easily formed at pH < 3, and goethite (α-FeOOH) is generated at pH > 5 (Acero et al., 2006; Liao et al., 2009). Many studies have found that Fe-containing secondary minerals are suitable in removing As pollution due to their great adsorption efficiency to As. For example, schwertmannites can remove significant fractions of As(III) (>97%) from contaminated water, and their As(III) sorption behavior was strongly affected by the different synthesis pathways (Paikaray et al., 2011). However, how microbial communities and the Fe/S redox reactions they catalyze affect Fe/S speciation has not yet been fully described. Also, the links between Fe/S speciation transformations and As fate have rarely been studied.

The water column system could be an effective way to control water levels (Tokunaga et al., 2018). In the present study, we used acidic water columns to evaluate the links above. To the water columns, we inoculated the mixture of the chemoautotrophic *Acidithiobacillus ferrooxidans (At. ferrooxidans)* and the heterotrophic *Acidiphilium acidophilum (Aph. acidophilum)* and added three energy substrates, Fe^2+^, S^0^, and glucose, as well as two concentrations of As(III) (2.0 and 4.5 mM). Through monitoring Fe/S/As speciation transformations and the dynamic change in the microbial community along the depths of the water columns, we comparatively investigated their correlations at three different depths of the water columns. These two strains are widely occurring together in AMD environments (Hedrich and Schippers, 2021) and have been commonly used to simulate the bioleaching of metal sulfide minerals under acidic conditions (Liu et al., 2011; Liu, 2013). This work could be valuable in elucidating As transformations mediated by microbially driven Fe/S redox in AMD environments.

## Materials and Methods

### Bacterial Strains and Culture Conditions

The strains *At. ferrooxidans* ATCC 23270 (NR_041888) and *Aph. acidophilum* ATCC 27807 (NR_036837) used in this study were provided by the Key Laboratory of Biometallurgy of the Ministry of Education of China, Changsha, China. The strain ATCC 23270 is an obligately autotrophic organism. It can use Fe^2+^, H_2_, S^0^, and other reduced sulfur compounds as energy substrates for growth under aerobic conditions and use H_2_ or S^0^ under anaerobic conditions by using Fe^3+^ as the electron acceptor. The strain ATCC 27807 is a facultative heterotrophic organism. It can use glucose and inorganic reducing sulfur compounds as energy substrates and grow aerobically or anaerobically with O_2_ or Fe^3+^ as the electron acceptor (Johnson and Bridge, 2002; Ohmura et al., 2002; Liu et al., 2011). In this experiment, we used the coculture of *At. ferrooxidans* and *Aph. acidophilum* as a mixed bacterial consortium.

Before the simulated column experiment, *At. ferrooxidans* and *Aph. acidophilum* were domesticated for several generations. *At. ferrooxidans* was cultured in 9K basal medium containing 44.7 g/L Fe^2+^ and 10 g/L S^0^, and *Aph. acidophilum* was cultured in 9K basal medium with 1 g/L glucose and 0.1 g/L Fe^3+^ with an initial pH of 2.5. The chemical components for 9K basal medium were as follows (per liter): (NH_4_)_2_SO_4_, 3 g; K_2_HPO_4_, 0.5 g; MgSO_4_⋅7H_2_O, 0.5 g; KCl, 0.1 g; and Ca(NO_3_)_2_, 0.01 g.

### Simulated Column Experiment

The simulated column experiment was performed in 10 self-made water columns (nine for the experiment group and one for the sterile control) with 1.15 m height, 0.12 m inside diameter, and 0.01 m thickness of the column wall (Supplementary Figure 1). For sampling at three depths of the columns (15, 55, and 105 cm), each column was equipped with three hollow tubes (1 mm inner diameter) on the inner wall. Before being added to each column of the experiment group, the sterilized 9K basal medium (11.9 L) containing 44.7 g/L FeSO_4_, 1 g/L Fe_2_(SO_4_)_3_, 10 g/L S^0^, and 1 g/L glucose was mixed well with 0, 3.09, or 6.96 g analytical-grade NaAsO_2_ in a sterile beaker, with a final As(III) concentration of 0, 2.0, or 4.5 mM, respectively. The three resulting media were adjusted to pH 2.5 with 1 M H_2_SO_4_ and inoculated with the mixture of *At. ferrooxidans* (2.5 × 10^7^ cells/ml) and *Aph. acidophilum* (2.5 × 10^7^ cells/ml) and then poured into the water columns (each up to 1.05 m). The water columns were then sealed by sterile vented films (0.2–0.3 μm) and cultured under the static condition at room temperature for 144 days. For each concentration of As(III), the water column experiment was performed in triplicate. The columns with 0, 2.0, and 4.5 mM NaAsO_2_ added were assigned to Col_0, Col_2.0, and Col_4.5, respectively. The uninoculated water column control was filled with the above medium without NaAsO_2_, and it was used to measure the oxygen concentration at three depths of the column at days 0 and 60.

During cultivation, pH, ORP, DO, and concentrations of Fe^3+^, Fe^2+^, As(III), As(V), and SO_4_^2–^ were monitored for the experiment group columns at three different depths of 15, 55, and 105 cm, representing the top, middle, and bottom sites and assigned as Col_/Top_, Col_/Mid_, and Col_/Bot_, respectively. In order to avoid disturbance to the solution, the DO electrodes were fixed at different depths (15, 55, and 105 cm) before the experiment.

### Analytical Methods

During the experiment, the solution samples (2 ml for each) were taken out from the three depth sites by disposable sterile syringes through the hollow tubes at 4-, 5-, or 10-day intervals to determine cell density, concentrations of SO_4_^2–^, total Fe (TFe), Fe^3+^, total As (TAs), and As(III). The total cell density was determined by direct counting with a blood corpuscle counter (XB-K-25). [SO_4_^2–^] was measured by the barium sulfate turbidimetric colorimetric method according to Zhu et al. (2011). [TFe] and [Fe(III)] were determined by 5-sulfosalicylic acid spectrophotometry according to Karamanev et al. (2002). The Fe^2+^ concentrations were obtained by subtracting [Fe^3+^] from [TFe]. [As(III)] was analyzed by an atomic fluorescence spectrometer (AFS) (PS Analytical Ltd., Kent, United Kingdom). [TAs] was measured by an inductively coupled plasma-atomic emission spectroscopy (ICP-AES) (IRIS Intrepid II XSP, Thermo Fisher Scientific, United States). The As(V) concentrations were obtained by subtracting [As(III)] from [TAs]. The pH, ORP, and DO values of the solution were monitored *in situ* by a pH meter (PHS-3C), a platinum electrode with an Hg/HgCl_2_ reference electrode, and a DO meter (AZ-8403), respectively. The above data were analyzed statistically with Excel 2015 and SPSS 20.0 software and presented in terms of the mean value with the standard deviation (SD) as the error bar from triplicate cultures (*n* = 3).

Samples for bacterial composition analysis were collected by centrifugation of 20–50 ml solution at three depths of the columns. The V4–V5 region of the 16S rRNA gene was sequenced using an Illumina Nova 6000 platform (Illumina, San Diego, United States) and 250 bp paired-end reads by Magigene Co., Ltd., Guangzhou, China. The raw sequence data were processed with the Cutadapt software to obtain the paired-end clean reads. Then the paired-end clean reads were merged using usearch-fastq_mergepairs according to the relationship of the overlap between the paired-end reads, and the spliced sequences were raw tags. And Fastp was used to control the quality of the raw data by using sliding windows to obtain paired-end clean tags. Subsequently, the remaining sequences were clustered into operational taxonomic units (OTUs) with the UPARSE algorithm (Edgar, 2013). For each representative sequence, the SILVA database was used to annotate taxonomic information by the usearch-sintax with a confidence threshold of 0.8 (Quast et al., 2013). The bacterial abundance of *At. ferrooxidans* and *Aph. acidophilum* was obtained by adding all OTUs of the genus *Acidithiobacillus* and *Acidiphilium*, respectively. The bacterial composition of *At. ferrooxidans* or *Aph. acidophilum* was obtained by multiplying the total cell density by the percentage of the abundance of *At. ferrooxidans* or *Aph. acidophilum*. Raw reads are publicly available at the NCBI Sequence Read Archive under BioProject PRJNA783759^1^.

The suspended matter formed at the top, and the residues at the bottom of the water columns were collected at different times for phase compositions and Fe/S/As speciation analyses by synchrotron radiation X-ray diffraction (SR-XRD), FT-IR spectroscopy, X-ray photoelectron spectroscopy (XPS), and X-ray absorption near-edge structure (XANES) spectroscopy. In detail, the suspended matter formed at the top of the columns was collected when it was visibly formed, while the residues at the bottom were collected on days 8, 40, 74, 100, and 140. The samples for these analyses were dried using a DFZ6050 vacuum drying oven. The SR-XRD was performed on the BL14B1 beamline at the Shanghai Synchrotron Radiation Facility (SSRF), Shanghai, China, with a step of 0.01° and a dwell time of 0.5 s at each point (Xia et al., 2018). The FT-IR spectra were detected using a Fourier transform spectrometer (Nexus 670, Nicolet, United States) in transmittance mode. The FT-IR spectra were recorded over 500–4,000 cm^–1^ after mixing 0.9 mg of each sample with 80 mg of KBr and pressing the mixture into a pellet (Liu et al., 2021). The XPS spectra were collected using a Thermo Fisher Scientific K-Alpha+ X-ray photoelectron spectrometer using monochromatized Al Kα (1,486.6 eV) radiation as the excitation radiation. All photoelectron binding energies were referenced to C 1 s adventitious contamination peaks set at 285.0 eV. The Fe L-edge and S K-edge XANES analyses were carried out on the 4B7B beamline and 4B7A beamline, respectively, at the Beijing Synchrotron Radiation Facility (BSRF), Beijing, China (Xia et al., 2018). The Fe L-edge XANES spectra data were recorded in total electron yield (TEY) mode at step widths of 0.5 eV from 690 to 700 eV and 0.1 eV from 700 to 928 eV. The S K-edge XANES spectra data were recorded in fluorescence mode at ambient temperature and scanned at a step width of 0.2 eV from 2,450 to 2,520 eV. The XANES spectra were normalized to the maximum of the adsorption jump and fitted by the linear combination of reference spectra with the IFEFFIT program as previously described (Ravel and Newville, 2005).

## Results

### Cell Growth and Depth Distribution of *Acidithiobacillus ferrooxidans* and *Acidiphilium acidophilum*

**FIGURE 1:**
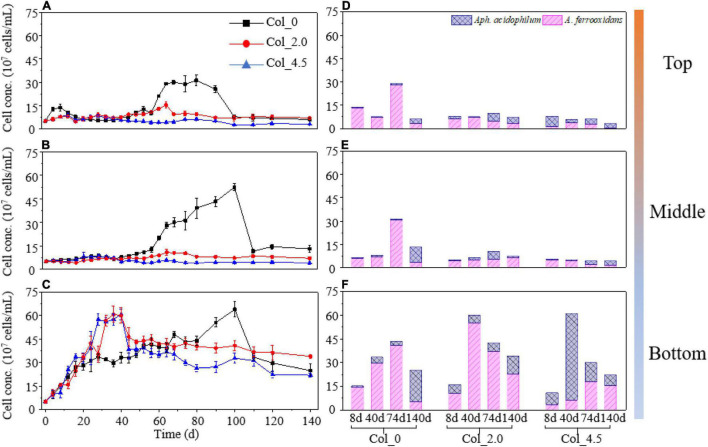
Cell density **(A–C)** and bacterial composition **(D–F)** for the top **(A,D)**, middle **(B,E)**, and bottom **(C,F)** of the water columns without (Col_0) and with additions of 2.0 mM (Col_2.0) and 4.5 mM (Col_4.5) As(III). The initial cell density of *At. ferrooxidans* and *Aph. acidophilum* was the same, i.e., 2.5 × 10^7^ cells/ml.

Cell growth and depth distribution of *At. ferrooxidans* and *Aph. acidophilum* varied greatly at three different depths of the water columns without and with additions of 2.0 and 4.5 mM As(III) (Figures 1A–C). For the cases with As(III) added, the cell density at the bottom (Figure 1C) was significantly higher than that at the top and middle (Figures 1A,B) during the whole cultivation; while when no As(III) was added, the cell densities at the top, middle, and bottom showed similar trends, i.e., gradually increased and then rapidly decreased, and the highest value of the cell density followed the order Col_/Mid_ ≈ Col_/Bot_ > Col_/Top_. By comparing the three cases, it was found that the additions of 2.0 and 4.5 mM As(III) resulted in a significant decrease in the growth rate at the top and middle on days 0–16 and 68–100 (Figures 1A,B), but a significant increase at the bottom on days 24–44 (Figure 1C).

As shown in Figures 1D–F and Supplementary Table 1, when no As(III) was added, the cell density of *At. ferrooxidans* in the co-culture was much higher than that of *Aph. acidophilum* on days 8, 40, and 74 at all three depths of the water columns, indicating that *At. ferrooxidans* was dominant therein, while the proportion of *At. ferrooxidans* gradually decreased from the top to the bottom on day 140, suggesting that the other species, i.e., *Aph. acidophilum*, gradually became dominant. The proportion of *At. ferrooxidans* for the case with 2.0 mM As(III) added, i.e., Col_2.0, at the bottom was higher than that for the case with 4.5 mM As(III) added, i.e., Col_4.5; whereas the cell density of *At. ferrooxidans* increased from days 8 to 40 and then gradually decreased until day 140 for Col_2.0_/Bot_ and for Col_4.5_/Bot_, the cell density of *Aph. acidophilum* showed similar changes.

### Changes in pH and Major Ion Concentrations in the Solution

**FIGURE 2:**
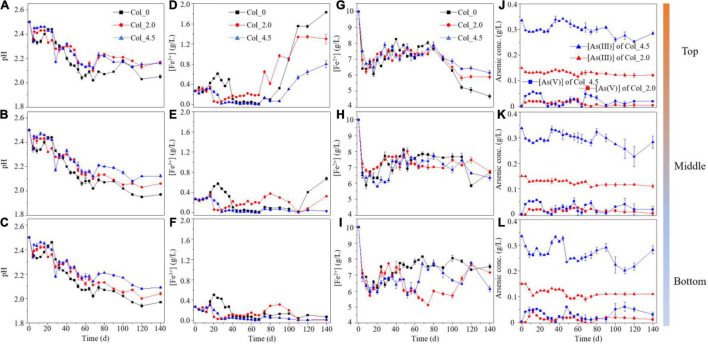
The curves of pH **(A–C)**, [Fe^3+^] **(D–F)**, and [Fe^2+^] **(G–I)** in the solutions at the top **(A,D,G,J)**, middle **(B,E,H,K)**, and bottom **(C,F,I,L)** of the water columns during cultivation without (Col_0) and with additions of 2.0 mM (Col_2.0) and 4.5 mM (Col_4.5) As(III), and the curves of [As(III)] and [As(V)] **(J–L)** with additions of 2.0 and 4.5 mM As(III). Error bars represent SD, *n* = 3.

Figure 2 shows the changes in pH and major ion concentrations ([Fe^2+^] and [Fe^3+^], [As(III)] and [As(V)]) in the solution for three cases at different depths. It can be seen from Figures 2A–C that the pH values for all cases gradually decreased and that the pH values for the case without As(III) was lower than that with As(III) added on days 30–140, likely due to the inhibition of microbial activity by the added As(III). In the comparison of pH values for the cases with As(III) added, the inhibition effect increased with As(III) concentration added at the middle and the bottom on days 0–32 and 64–140 and at the top on days 0–32 and 90–110, while there was no significant difference for that at other times.

The changes in [Fe^3+^] (Figures 2D–F) for three cases showed similar trends on days 0–68, indicating that the Fe redox reactions were not much different for all depths of each case, but the [Fe^3+^] on days 72–140, however, significantly increased at the top compared with that at the middle and bottom, indicating that differentiation of Fe redox gradually occurred at different depths, which was also consistent with the changes in ORP values in the solution (Supplementary Figure 2). The [Fe^2+^] (Figures 2G–I) and [SO_4_^2–^] (Supplementary Figure 3) showed similar trends and changed little for different depths in the case without As(III), while they changed more significantly for that with As(III) added, especially [Fe^2+^] on days 48–60 for Col_4.5_/Bot_ and on days 48–100 for Col_2.0_/Bot_, as well as [SO_4_^2–^] on days 64–68 for Col_4.5_/Bot_ and on days 56–110 for Col_2.0_/Bot_, which was significantly lower than that for the top and middle of Col_4.5 and Col_2.0, probably due to the formations of Fe- and/or SO_4_^2–^-containing precipitates.

The [As(III)] (Figures 2J–L) at three different depths of Col_2.0 and Col_4.5 showed a general slightly decreasing trend throughout the time course of the experiment. In the comparison of [As(III)] and [As(V)] at each depth, the results showed that, for the case with 2.0 mM As(III) added, the As(III) at the top, middle, and bottom accounted for 92.1, 90.6, and 90.8% (on average) of the total As content, while for the case with 4.5 mM As(III) added, it accounted for 93.7, 92.8, and 92.0%, respectively. This result showed that As was mainly present in the solution in the form of As(III), indicating that only < 10% As(III) was oxidized to As(V).

### FT-IR and X-Ray Photoelectron Spectroscopy Analyses of Surface Suspended Matter

**FIGURE 3:**
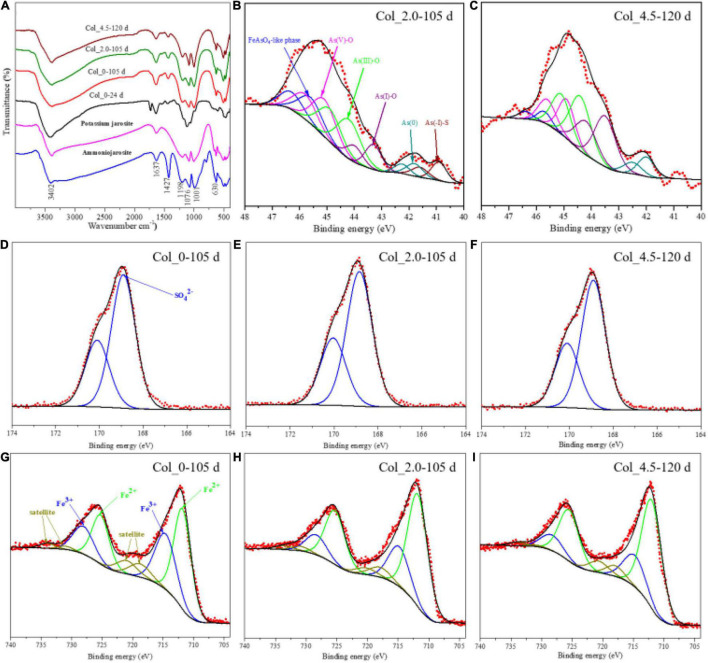
FT-IR spectra **(A)** and XPS spectra of the surface As **(B,C)**, S **(D–F)**, and Fe **(G–I)** for the surface suspended matter at 24/105, 105, and 120 days of the water columns without (Col_0) and with additions of 2.0 mM (Col_2.0) and 4.5 mM (Col_4.5) As(III), respectively.

During cultivation, suspended matter formed on the surface of the culture medium in three cases, but the time for forming such matter was quite different, i.e., on day 24 when no As(III) was added and on days 105 and 120 for the cases with the additions of 2.0 and 4.5 mM As(III), respectively (Supplementary Figure 4). The results of FT-IR spectra for the suspended matter (Figure 3A) show a series of bands of potassium jarosite and ammonium jarosite at 3,402, 1,637, 1,427, 1,198, 1,076, 1,001, and 630 cm^–1^, indicating that the suspended products on the surface were a mixture of potassium jarosite and ammonium jarosite.

The As 3d XPS spectra and their fitted results are shown in Figures 3B,C and Supplementary Table 2, respectively. It is known that XPS spectra are suitable for characterizing the components on the topmost surface (typically < 10 nm) of the target matter. It can be seen from both Figures 3B,C and Supplementary Table 2 that the As species on the surface of suspended matter were composed of As(-I), As(0), As(I)-O, As(III)-O, As(V)-O, and FeAsO_4_⋅2H_2_O-like phase (Fantauzzi et al., 2011), which are assigned by the binding energies at 40.9 ± 0.1, 41.8 ± 0.2, 43.2 ± 0.3, 44.1 ± 0.3, 45.1 ± 0.3, and 45.6 eV, respectively. The surface As species compositions for the two cases with As(III) added were quite different, e.g., 7.89% As(-I), 6.17% As(0), 12.07% As(I)-O, 29.95% As(III)-O, 25.77% As(V)–O, and 18.15% FeAsO_4_⋅2H_2_O for the case with 2.0 mM As(III) added, while for the case with 4.5 mM As(III) added, they changed to 9.66% As(0), 32.17% As(I)–O, 33.83% As(III)–O, 19.51% As(V)-O, and 4.83% FeAsO_4_⋅2H_2_O. The surface iron species were composed of Fe(III) and Fe(II), according to Figures 3G–I. According to the fitting results (Supplementary Table 3), the proportion of Fe(III) species was 40.39% for the case without As(III) added, while it was 27.51 and 24.76% for the cases with 2.0 and 4.5 mM As(III), respectively. The S 2p XPS spectra (Figures 3D–F) were only fitted with one doublet peak of SO_4_^2–^ at 168.5 ± 0.3 eV, which is consistent with the FT-IR results assigning the SO_4_^2–^-containing jarosites in the suspended matter (Figure 3A).

### Fe/S/As Speciation Analyses of the Bottom Residues

**FIGURE 4:**
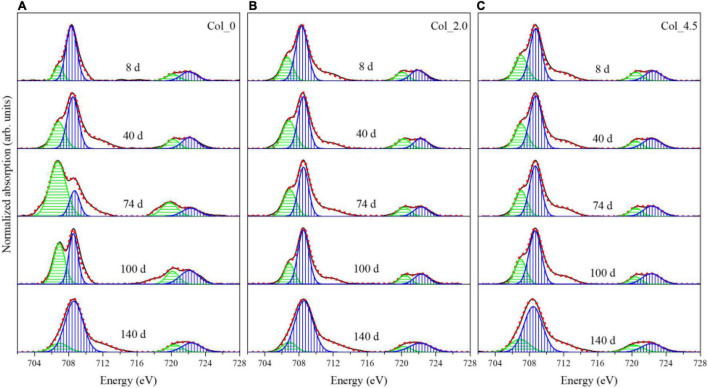
Normalized Fe L-edge XANES spectra for the bottom residues during cultivation without **(A)** and with additions of 2.0 mM **(B)** and 4.5 mM **(C)** As(III), where the green and blue bands represent Fe(II) and Fe(III) species, respectively.

**FIGURE 5:**
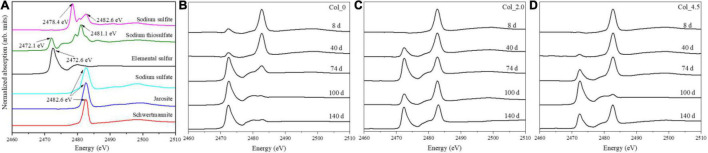
S K-edge XANES spectra for the reference samples **(A)**, and the bottom residues during cultivation without **(B)** and with additions of 2.0 mM **(C)** and 4.5 mM **(D)** As(III).

**FIGURE 6:**
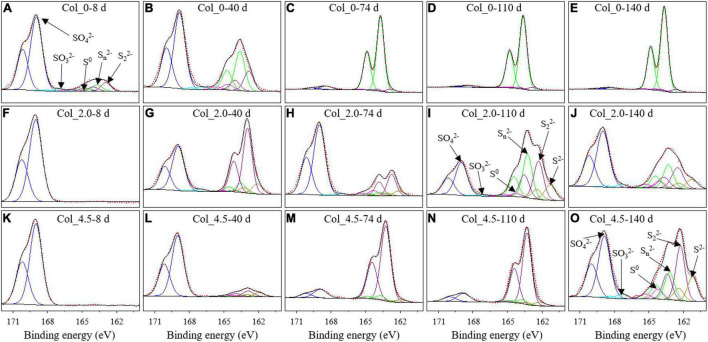
XPS spectra for the surface S of the bottom residues during cultivation without **(A–E)** and with additions of 2.0 mM **(F–J)** and 4.5 mM **(K–O)** As(III) on days 8 **(A,F,K)**, 40 **(B,G,L)**, 74 **(C,H,M)**, 110 **(D,I,N)**, and 140 **(E,J,O)**.

**FIGURE 7:**
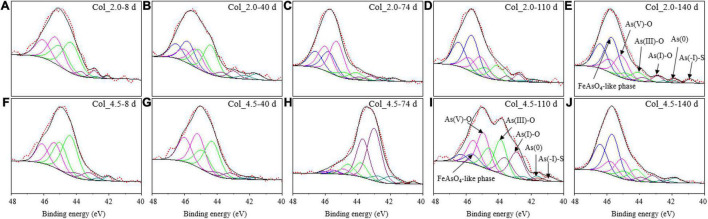
XPS spectra for the surface As of bottom residues during cultivation with additions of 2.0 mM **(A–E)** and 4.5 mM **(F–J)** As(III) on days 8, 40, 74, 110, and 140.

During cultivation, the phase composition of the bottom residues was mainly composed of S^0^ and very few jarosites with little change according to the XRD analysis (Supplementary Figure 5). However, the Fe/S/As speciation analysis results show that the complex Fe/S/As speciation transformations of the bottom residues occurred (Figures 4–7).

The Fe L-edge XANES spectra (Figure 4) and the fitted results (Supplementary Table 4) for the bottom residues show that both the L_3_-edge and L_2_-edge contain the Fe(II) and Fe(III) absorption peaks, i.e., E_a_ (706.8 eV) and E_b_ (708.3 eV) for L_3_-edge and E_c_ (720.1 eV) and E_d_ (722.1 eV) for L_2_-edge (Mikhlin and Tomashevich, 2005). When no As(III) was added, the proportion of Fe(II) gradually increased up to 77.62% of the total Fe species on day 74 and then decreased to 14.70% on day 140. For the case with 2.0 mM As(III) added, the proportion of Fe(II) species gradually increased up to 40.09% on day 40 and then decreased to 15.6% on day 140, while for the case with 4.5 mM As(III) added, the proportion of Fe(II) species remained about 50–54% of the total Fe before day 74 and then gradually decreased to 10.71% on day 140. These results suggested that the additions of As(III) can clearly inhibit Fe reduction.

The S K-edge XANES spectra are shown in Figure 5, with the fitted results being given in Supplementary Table 5. In comparison with the reference samples (Figure 5A), it was observed that when no As(III) was added, the intensity of the peak at 2,482.6 eV for SO_4_^2–^ gradually decreased, while the intensity of the peak at 2,472.6 eV for S^0^ gradually increased, and that there was a slight characteristic peak at 2,479.6 eV associated with sodium thiosulfate on days 74–140 (Figure 5B). For the case with 2.0 mM As(III) added, the proportion of SO_4_^2–^ species gradually decreased to 33.0% on days 8–74 and then increased to 70.2% on day 100 and decreased to 47.1% on day 140, while the proportion of S^0^ species followed opposite trends; and 19.0% S_2_O_3_^2–^ species were also detected on day 140 (Figure 5C). For the case with 4.5 mM As(III) added, the proportion of SO_4_^2–^ species gradually decreased to 3.2% and that of S^0^ species increased to 93.5% on days 8–100, and then the proportion of SO_4_^2–^ species increased to 58.7% and that of S^0^ species decreased to 24.1% on day 140; there was also 17.2% S_2_O_3_^2–^ species on day 140 (Figure 5D).

The S 2p XPS spectra for the bottom residues (Figure 6) further show that the surface sulfur species are composed of S^2–^ (162.1 eV), S_2_^2–^ (As-S, 163.1 eV), S_n_^2–^ (163.8 eV), S^0^ (164.6 eV), SO_3_^2–^ (166.5 eV), and SO_4_^2–^ (168.8 eV). The fitted results (Supplementary Table 6) showed that no As(III) added led to a decrease in SO_4_^2–^ and an increase in S_n_^2–^, with a little proportion of S^0^ and 2.13–2.19% SO_3_^2–^ on days 8–40. Additionally, no S^2–^ was detected throughout the whole experiment. While for the two cases with As(III) added, SO_4_^2–^ was the only sulfur speciation on day 8, and on day 40, S^2–^, S_2_^2–^, and S_n_^2–^ also appeared. On days 74–140, S_2_^2–^ became the major sulfur species for the case of 4.5 mM As(III). These results suggested that compared with those obtained from samples with no As(III) addition, the sulfur components in the bottom residues for the cases with As(III) added became more complex with the formation of more S_2_^2–^ and S^2–^, instead of S_n_^2–^.

The As 3d XPS spectra (Figure 7) and fitted results (Supplementary Table 7) for the bottom residues of the cases with As(III) added show that the As species on the surface of bottom residues were composed of the FeAsO_4_⋅2H_2_O-like phase (45.6 ± 0.1 eV), As(V)-O (45.1 ± 0.3 eV), As(III)-O (44.1 ± 0.3 eV), As(I)-O (43.2 ± 0.2 eV), and As(0) (41.8 ± 0.2 eV) and a little As(-I)-S (40.9 ± 0.1 eV), similar to that of the suspended matter. It was noted that for the two cases with As(III) added, the changes in the composition of As species were different during days 8–140, though similar in the main components As(III)-O and As(V)-O species on day 8 and FeAsO_4_ and As(V)-O on day 140. For instance, the FeAsO_4_ species appeared on day 40 for the case with 2.0 mM As(III) added, much earlier than that with 4.5 mM As(III) added; the variation trends of As(V)-O species showed the opposite trends. In addition, the proportion of As(I) increased up to 64.45% on day 74 and then decreased to 6.45% on day 140 for the case of 4.5 mM As(III) added, while that for the case with 2.0 mM As(III) added remained low (∼5–7%).

## Discussion

### Bacterial Growth and Depth Distribution of Microbial Communities Responding to As(III) Toxicity

It has been widely reported that As(III) could significantly inhibit bacterial growth and the Fe/S oxidation activities (Hallberg et al., 1996; Páez-Espino et al., 2009). In the present study, we further found that in the simulated water columns, both the bacterial growth and composition were affected not only by the additions of As(III) but also by the depths of the water columns (Figure 1).

For the columns with additions of As(III), the cell growth was significantly inhibited at the top and middle (Figure 1A), likely due to the toxicity of dissolved As(III) to bacterial cells. On the other hand, irrespective of the As(III) addition (or a lack of it), the cell growth at the bottom of the water columns quickly entered the exponential phase, which is much different from the top and middle, where the cell growth exhibited typical growth curves with about 40 days of lag phase. The promotion of bacterial growth at the bottom of the columns with the addition of As(III) was probably due to the formation of Fe/As/S-containing precipitates, FeAsO_4_⋅2H_2_O, and jarosites (Figures 4–7), which can adsorb to or coprecipitate with As species. These processes could significantly mitigate the harmfulness of As(III) and reduce the Fe^3+^ concentration and thus mitigate the inhibited feedback of Fe(III) to the ferrous-oxidizing enzyme activity (Hare et al., 2020; Zhang et al., 2020). Moreover, the cells detected at the bottom showed somewhat As adsorption according to the results of FT-IR (Supplementary Figure 8), which could also reduce As harmfulness and thus favor cell growth (Zhang et al., 2021).

The bacterial compositions of *At. ferrooxidans* and *Aph. acidophilum* during cultivation were also significantly affected by the additions of As(III) (Figures 1D–F) and the depths of the water columns. When no As(III) was added, *At. ferrooxidans* was dominant during the most experimental time at the top, middle, and bottom of the water columns. However, for both cases with As(III) added, *At. ferrooxidans* dominated at the top and middle of the columns, while in the case with a higher concentration of As(III), *At. ferrooxidans* decreased and became less abundant than *Aph. acidophilum* at the bottom. This may be due to the differences in the energy substrate depth distribution along with the water columns and the metabolism between the two bacteria. Considering the difference in the solubilities of Fe and S substrates and the phenomenon that S^0^ basically deposited at the bottom of the columns during the most experimental time, it was concluded that there was no difference in the glucose distribution but that there is an obvious difference in Fe and S energy substrate distributions along with the depth of the water columns. Therefore, at the top and middle of the columns, there were major Fe^2+^ and slight soluble reducing sulfur compounds, S_n_^2–^, as shown by the results of S K-edge XANES and S 2p XPS (Figures 5, 6). While at the bottom, there were large amounts of S^0^ distributed, besides Fe^2+^. Based on the above experimental phenomena and inferences, it was speculated that *At. ferrooxidans* grew majorly on soluble Fe^2+^ at the top and middle, and at the bottom, it grew majorly on S^0^ deposited, while *Aph. acidophilum* grew on glucose at the top and middle and used S^0^ and glucose at the bottom of the water columns. Furthermore, both bacteria could use O_2_ and Fe^3+^ as electron acceptors; however, DO (Supplementary Figure 6) and Fe^2+^ (Figures 2G–I) concentration measurements indicated that there were aerobic conditions and no lack of Fe^2+^ for all three depths of the columns for most of the experimental time; thus, it was very likely that the microbial Fe^2+^ oxidation (as opposed to Fe^3+^ reduction) played a major role at the top and middle of the columns. Moreover, the higher As(III) concentration may obviously inhibit the growth of *At. ferrooxidans*. It implies that there were differences in As resistance between the two strains, which may be the reason for the major change in the bacterial distribution for the case with a higher As(III) concentration (Dopson and Johnson, 2012; Mirete et al., 2017).

### Iron and Sulfur Speciation of the Simulated Water Column

The variations of physicochemical parameters and the concentrations of dissolved Fe and S at different depths of the water columns were different. The pH values (Figure 2A) were gradually decreasing at three depths of all the water columns, which can be mainly caused by sulfur bio-oxidation (Eq. 1) and partly by the formation of jarosites (Eq. 2) (Xia et al., 2010; He et al., 2012; Zhang et al., 2021). The decrease in pH values slowed down with the additions of As(III), and the slowdown decreased with more additions of As(III), indicating the inhibition of As(III) to sulfur bio-oxidation and/or the formation of jarosites. Moreover, the decrease in the pH values to some extent depended on different depths of the water columns and cultivation time, which is probably related to the evolution of the bacterial composition of *At. ferrooxidans* and *Aph. acidophilum* during cultivation.

Fe^2+^ bio-oxidation was more inhibited with the increase of [As(III)] and solution depths (Figure 2B). The [Fe^3+^] remained very low at the bottom sites for all the water columns, which was likely related to the low Fe^2+^ bio-oxidation activity and the Fe^3+^ reduction by *Aph. acidophilum* under microaerobic conditions (Supplementary Figure 6). The changes in the potential of the solution also indicated that the obvious inhibition of Fe^2+^ oxidation occurred at the middle and bottom of the water columns. Generally speaking, changes in [Fe^3+^], [Fe^2+^], [SO_4_^2–^], and potential in solutions at the three water column depths seemed to be very complex (Figures 2D–I and Supplementary Figures 2, 3), which may be due to the complex interactions (adsorption, complexation, and coprecipitation) between Fe, S, and As species and the formation of jarosites (Eq. 2), scorodite (Eq. 3), and other uncertain complexes.


(1) $$\text{S}+1.5\,O_{2}+{\text{H}}_{2}\,\text{O}\to 4\,H^{+}+S\,O_{4}^{2-}$$

(2) $${\text{M}}^{+}+3\,F\,{\text{e}}^{3+}+2\,S\,{\text{O}}_{4}^{2-}+6\,H_{2}\,\text{O}\to {\text{MFe}}_{3}\,{\left({\text{SO}}_{4}\right)}_{2}\,{\left(\mathrm{OH}\right)}_{6}+6\,H^{+}$$

where M is a monovalent cation, such as NH_4_^+^, K^+^, Na^+^, and H_3_O^+^.


(3) $${\text{Fe}}^{3+}+{\text{AsO}}_{4}^{3-}\to \text{FeAs}\,O_{4}$$

At the top of the water columns, the Fe^3+^ concentrations were relatively high (Figure 2D) in the middle and late stages of cultivation due to the apparent Fe^2+^ bio-oxidation. The Fe^3+^ could react with other monovalent cations and SO_4_^2–^ in the solution to form potassium and ammonium jarosites suspended on the surface of the solution (Figure 3A), which was in accordance with the appearance of the yellow surface suspended matter in the acidic environment of AMD (Supplementary Figure 4). The Fe 2p signal (Figures 3G–I) further showed that the additions of As(III) could inhibit the Fe^2+^ oxidation of the suspended solid matter at the top of the water columns. The higher the As(III) concentration, the more the inhibition to Fe^2+^ oxidation. Therefore, the added As(III) could delay the formation of jarosites on the solution surface (Supplementary Figure 9). The formation of amorphous FeAsO_4_⋅2H_2_O occurred for the case of 2.0 mM As(III) (Figure 3B), while its formation was inhibited by the addition of 4.5 mM As(III) (Figure 3C). This indicated that the high concentration of As(III) could inhibit As oxidation as well as the formation of FeAsO_4_⋅2H_2_O.

The Fe and S speciation changes at the bottom of the water columns were more complex than those at the top. When no As(III) was added, S oxidation (Figure 5B) and Fe reduction (Figure 4A) occurred in the early stage of the cultivation, and Fe oxidation was dominant after day 74, while the S speciation on the surface of the residues did not change significantly (Figure 6A). However, the addition of 2.0 mM As(III) inhibited Fe reduction (Figure 4B) but significantly promoted sulfur oxidation (Figure 5C) and produced more diverse sulfide species (Figure 6B) in the late experimental stage. When 4.5 mM As(III) was added, the composition change of S speciation was similar to the case with 2.0 mM As(III) added, but the promotion effect of sulfur oxidation was lower, while Fe reduction was promoted at the initial experimental stage (Figure 4C) and the proportion of Fe(II) began to decrease after day 100. These results indicated that Fe^3+^ reduction mediated by glucose or S oxidation occurred as a more likely process at the bottom of the water columns (Eqs 4, 5), with the S^0^ opening the ring and activating in contact with the outer membrane protein thiol group (P-SH, where P presents protein) on the cell membrane to form the polysulfide (Eqs 6, 7) (Rohwerder and Sand, 2003; Xia et al., 2013; Liu et al., 2015). It could be derived that *At. ferrooxidans* accounted mainly for S/Fe bio-oxidation at the bottom of the water columns when no As(III) was added, while for the cases with As(III) added, *Aph. acidophilum* increased obviously, and for the case with 4.5 mM As(III) added, it even became dominant in a certain period of cultivation with S_2_^2–^, instead of S_n_^2–^, as the main component in the bottom residues. It indicated that the bacterial distribution had an important effect on the Fe/S redox reactions.


(4) $${\text{C}}_{6}\,{\text{H}}_{12}\,{\text{O}}_{6}+24\,F\,{\text{e}}^{3+}+6\,H_{2}\,\text{O}\to 6\,C\,{\text{O}}_{2}+24\,F\,{\text{e}}^{2+}+24\,H^{+}$$

(5) $$12\,{\text{Fe}}^{3+}+2\,{\text{S}}^{0}+8\,{\text{H}}_{2}\,\text{O}\overset{\text{microbe}}{⟶}12\,{\text{Fe}}^{2+}+16\,\text{H}+2\,S\,O_{4}^{2-}$$

(6) $${\text{S}}_{8}+\text{P}-\text{SH}\to \text{P}-{\text{SS}}_{8}\text{H}\to \to \text{P}-{\text{S}}_{\text{m}}\text{H}\left(\text{m}\geq 9\right)$$

(7) $$\text{P}-{\text{S}}_{\text{m}}\text{H}+\text{P}-\text{SH}\to \text{P}-\text{S}-{\text{S}}_{\text{m-n}}-\text{P}+{\text{S}}_{\text{n}}^{2-}+2{\text{H}}^{+}\left(\text{n}\geq 1\right)$$

### As Speciation Changes and Their Relationship With Fe/S Redox

The complex As speciation transformations occurred in the solution, in the suspended matter at the top, and in the residues at the bottom of the water columns. The [As(III)] in the solution changed in the range of 0.2–0.35 and 0.1–0.15 g/L for the Col_2.0 and Col_4.5, respectively, while [As(V)] changed in the range of 0–0.05 g/L in all water columns (Figures 2J–L). It implies that the oxidation of As(III) to As(V) occurred in all depths of the water columns, but the oxidation rate of As(III) was very low, where only < 10% of As(III) was oxidized. Moreover, some of the As species in the solution were transferred to the solid phases by adsorption to and coprecipitation with the Fe(III)-(hydro)oxides and jarosites (Wang and Zhou, 2012; Ahoranta et al., 2016). Such processes could be affected by the solution pH and ORP (Supplementary Figure 7), as well as the As forms occurring (Paikaray et al., 2011; Ahoranta et al., 2016). Furthermore, As adsorption by bacterial cells (Supplementary Figure 8) could also contribute to the change in the composition of As species in the solution (Gao et al., 2018; Zhang et al., 2021).

Of note, the As species composition of the suspended matter at the top and the residues at the bottom were quite different for the cases with the additions of 2.0 and 4.5 mM As(III) (Figures 3, 7). For the suspended matter at the top, the As(III) oxidation and the formation of FeAsO_4_⋅2H_2_O seemed to be inhibited by the higher concentration of As(III), which was evidenced by the decrease of As(V)-O and FeAsO_4_⋅2H_2_O contents when 4.5 mM As(III) was added (Figure 3). For the bottom residues of the two cases with As(III) added, the main components of As species changed gradually from As(III) to As(V) species, and the proportion of As(V) was about 74.2–77.5% on day 140 for both cases (Figure 7 and Supplementary Table 7). It implies that As(V) was more easily precipitated onto the solid phases. Moreover, the formation of FeAsO_4_⋅2H_2_O species at the bottom for the case with 4.5 mM As(III) added was later than that with 2.0 mM As(III) added, and more amounts of FeAsO_4_⋅2H_2_O species were produced with less addition of As(III). It is noteworthy that there were amounts of As(I)-O species and a little As(0) and As(-I)-S species detected on the solid residues at the bottom, indicating that the reduction of As occurred at the bottom.

According to previous studies, the above different As speciation transformations in the water columns could be mostly associated with Fe/S redox reactions (Karimian et al., 2018). In aerobic conditions, As(III) and As(V) were easily released with the bio-oxidation of arsenopyrite by Fe/S-oxidizing bacteria (Smedley and Kinniburgh, 2002; Wang et al., 2020). Under anoxic conditions, the bacterial reductions of As(V) and Fe(III) oxides could influence the redox cycling of As, and the adsorbed As(III) on the ferric hydroxide substrate could enhance microbial Fe reduction (Campbell et al., 2006; Smeaton et al., 2012). In the present study, the DO changed at different depths during cultivation (Supplementary Figure 6), resulting in different redox environments associated with the depth distribution of *At. ferrooxidans* and *Aph. acidophilum* as discussed above. According to the changes in As species with the pH and ORP values (Supplementary Figure 7), As mainly existed as H_3_AsO_3_ in the solution at pH 0–4 and ORP 0–360 mV, and it was difficult to oxidize to As(V). This was in accordance with the situations for As(III) additions, the oxidation rate of As(III) was low, and As(III) was dominant in the solutions at different depths (Figures 2J–L). Of note, in the present study, it was observed that the higher As(III) concentration could inhibit the Fe^2+^ bio-oxidation and the formations of amorphous FeAsO_4_⋅2H_2_O and jarosites at the top of the water columns (Figure 3). However, the As reduction was enhanced by the higher As(III) concentration at the bottom (Figure 7 and Supplementary Table 7). According to the previous studies, the oxidation of As(III) to As(V) and the formation of FeAsO_4_⋅2H_2_O could be chemically coupled to the Fe reduction process, and the reduction of As(V) was probably mediated by microbial Fe oxidation (Campbell et al., 2006). It can be derived that the additions of different concentrations of As(III) could affect the Fe/S (bio-)redox behaviors and the Fe/S/As speciation transformation and thus lead to the change in the depth profile of the bacterial community structure.

**FIGURE 8:**
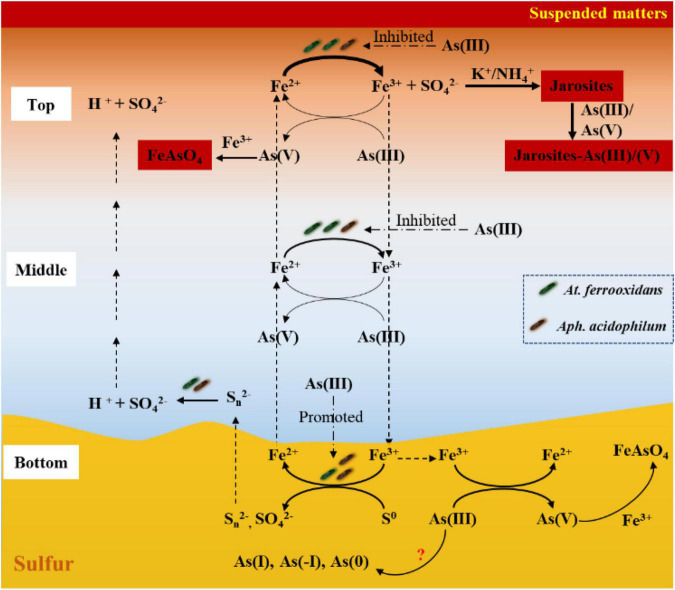
The diagram of the correlation between Fe/S/As speciation transformation and bacterial (*At. ferrooxidans* and *Aph. acidophilum*) depth distribution under different depths of the acidic water column. Dashed arrows show the migration of the dissolved ions, and solid arrows show the Fe/S/As redox reactions.

Based on the above discussion, the Fe/S/As speciation transformation and its relationship to the depth distribution of *At. ferrooxidans* and *Aph. acidophilum* in the simulated acidic water columns can be outlined (Figure 8). It shows that, on the top of the water columns, As(III) clearly inhibits bacterial growth and Fe^2+^ oxidation activity with more time required for the formation of jarosites. The higher the As(III) concentration, the more the inhibition to Fe^2+^ oxidation activity. The Fe^3+^ reduction coupled to As(III) oxidation occurs with the formation of As(V) and Fe^2+^; then the As(V) reacts with Fe^3+^ to form precipitate FeAsO_4_⋅2H_2_O, and the Fe^2+^ is recycled for further bio-oxidation. These processes can somewhat mitigate the inhibitory effects of As(III). In the middle of the water columns, Fe^2+^ bio-oxidation and Fe^3+^ reduction coupled to As(III) oxidation also occurred, but the Fe^2+^ bio-oxidation activity is more inhibited than that at the top, with no obvious precipitates formed. At the bottom of the water columns, besides the aforementioned similar Fe/As reactions, there also occurs sulfur oxidation coupled to Fe^3+^ reduction with the formation of Fe intermediates. These sulfur intermediates can diffuse to the middle and top of the water columns. In addition, As(III) can be reduced to As(I), As(0), and As(-I), which requires further study. These findings are of value for elucidating As fate and toxicity mediated by Fe/S speciation transformation driven by the microbial Fe/S redox in AMD environments.

## Conclusion

In the present work, the correlation between Fe/S/As speciation transformation and bacterial depth distribution was studied by using the acidic water columns. Such a correlation, as concluded in Figure 8, was apparently affected by both the additions of As(III) and the depths of the water columns and was affected differently by both the concentration and species of As. Though chemical reduction of Fe^3+^ coupled to As oxidation and Fe^2+^ bio-oxidation occurred at all three depths, the Fe/S/As speciation transformation and bacterial depth distribution showed obvious differences between the top and bottom of the water columns. It was apparently dependent on the distribution of energy substrates, Fe^2+^, reducing sulfur compounds and S^0^.

## Data Availability Statement

The datasets presented in this study can be found in online repositories. The names of the repository/repositories and accession number(s) can be found below: NCBI SRA database; accession numbers PRJNA783759, and SRR17048632 to SRR17048667.

## Author Contributions

Y-hZ: investigation, data curation, and writing—original draft. CW: investigation and validation. H-cL: conceptualization, methodology, and writing—review and editing. ZX: investigation and data curation. Z-yN: conceptualization and methodology. YL and J-lW: investigation. YY: conceptualization. W-sS: funding, conceptualization, and methodology. J-lX: funding, supervision, conceptualization, methodology, and writing—review and editing. All authors contributed to the article and approved the submitted version.

## Conflict of Interest

The authors declare that the research was conducted in the absence of any commercial or financial relationships that could be construed as a potential conflict of interest.

## Publisher’s Note

All claims expressed in this article are solely those of the authors and do not necessarily represent those of their affiliated organizations, or those of the publisher, the editors and the reviewers. Any product that may be evaluated in this article, or claim that may be made by its manufacturer, is not guaranteed or endorsed by the publisher.

## Footnotes

1
https://www.ncbi.nlm.nih.gov/bioproject/?term=783759
