# Supplementary material for: Correlation Between Fe/S/As Speciation Transformation and Depth Distribution of Acidithiobacillus ferrooxidans and Acidiphilium acidophilum in Simulated Acidic Water Column
Source: Front Microbiol. 2022 Feb 9;12:819804. doi: 10.3389/fmicb.2021.819804 (PMC8863614; doi:10.3389/fmicb.2021.819804)
Supplement: Supplementary file 1 [file Data_Sheet_1.docx]

# Supplementary Materials

*for*

**Correlation between Fe/S/As speciation transformation and depth distribution of *Acidithiobacillus ferrooxidans* and *Acidiphilium acidophilum* in simulated** **acidic water column**

Yu-hang Zhou^1^, Can Wang^1^, Hong-chang Liu^1^*, Zhen Xue^1^, Zhen-yuan Nie^1^, Yue Liu^1^, Jiao-li Wan^1^, Yu Yang^1^, Wen-sheng Shu^2^, Jin-lan Xia^1^*

^1^ Key Lab of Biometallurgy of Ministry of Education of China, School of Minerals Processing and Bioengineering, Central South University, Changsha, China

^2^ School of Life Science, South China Normal University, Guangzhou, China

*** Correspondence:** Hong-chang Liu, [hchliu2050@csu.edu.cn](mailto:hchliu2050@csu.edu.cn); Jin-lan Xia, [jlxia@csu.edu.cn](mailto:jlxia@csu.edu.cn)

The supplementary materials include 9 figures and 7 tables.

## Supplementary Figures


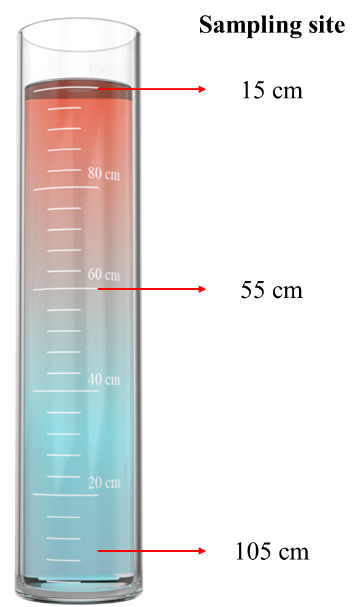


**Figure S1.** Schematic diagram of self-made water column. The water column made by acrylic is 1.15 m high, and 0.12 m inside diameters with 0.01 m thickness of the column wall.


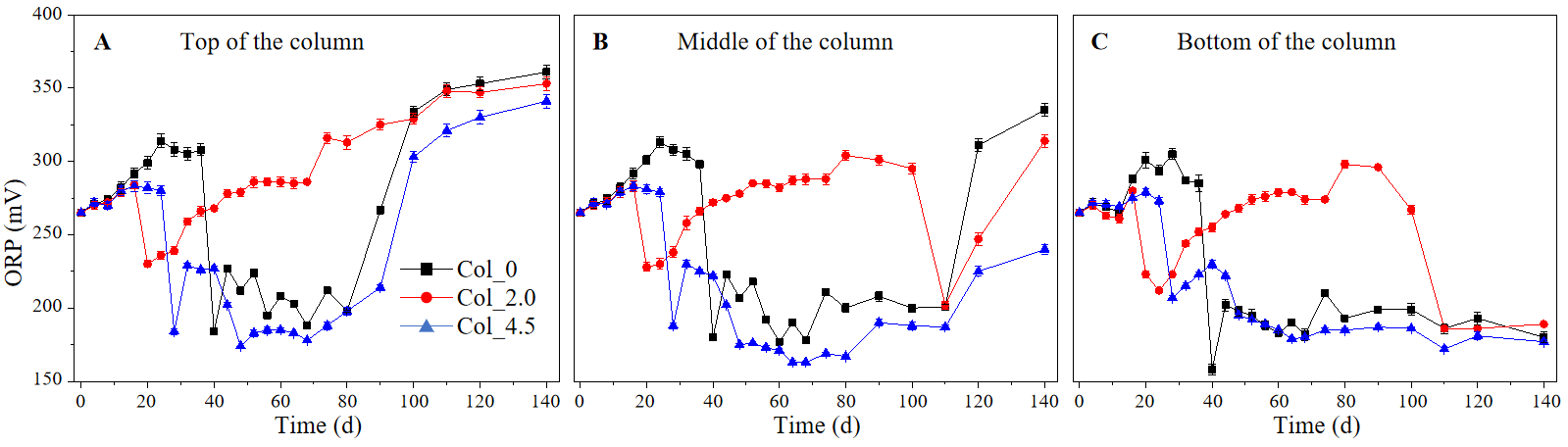


**Figure S2.** The curves of ORP in the solutions at the top (**A**), middle (**B**) and bottom (**C**) of water columns during cultivation without (Col_0) and with additions of 2.0 (Col_2.0) and 4.5 (Col_4.5) mM As(III).


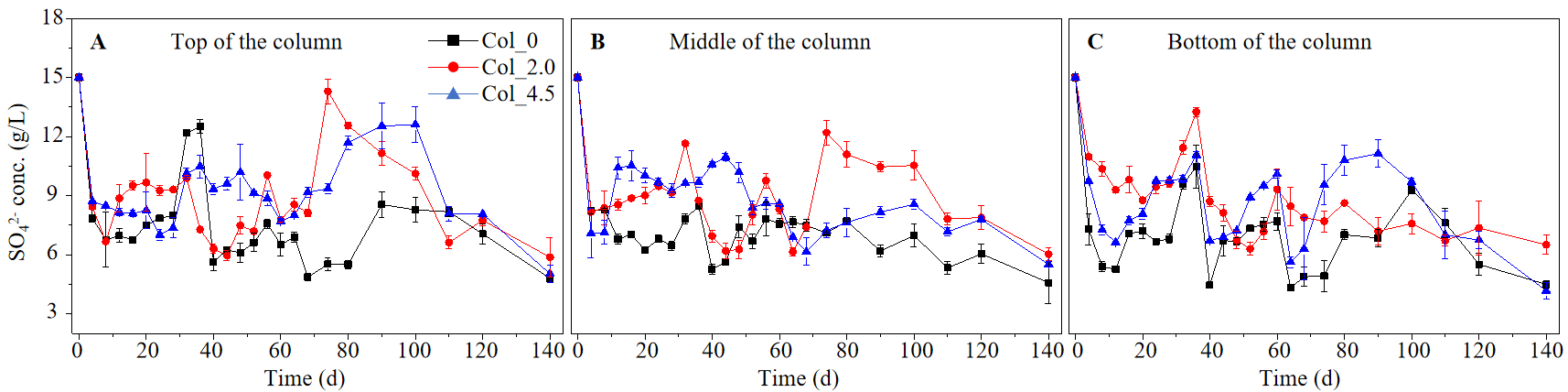


**Figure S3.** The change of [SO_4_^2-^] in the solutions at the top (**A**), middle (**B**) and bottom (**C**) of water columns during cultivation without (Col_0) and with additions of 2.0 (Col_2.0) and 4.5 (Col_4.5) mM As(III).


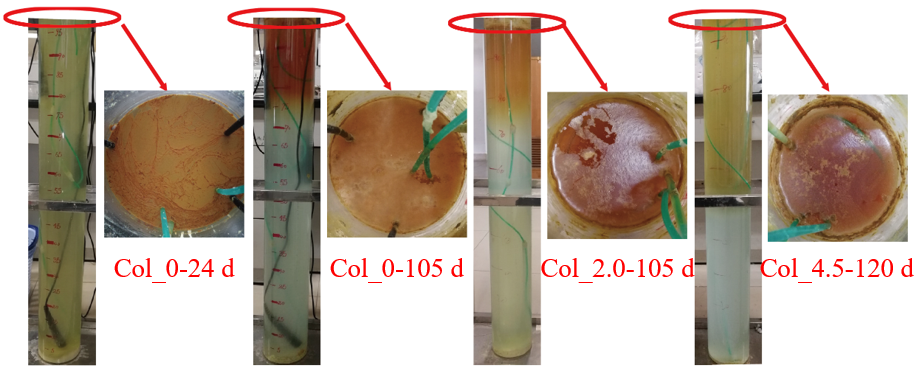


**Figure S4.** The images of surface suspended matter for Col_0, Col_2.0 and Col_4.5.


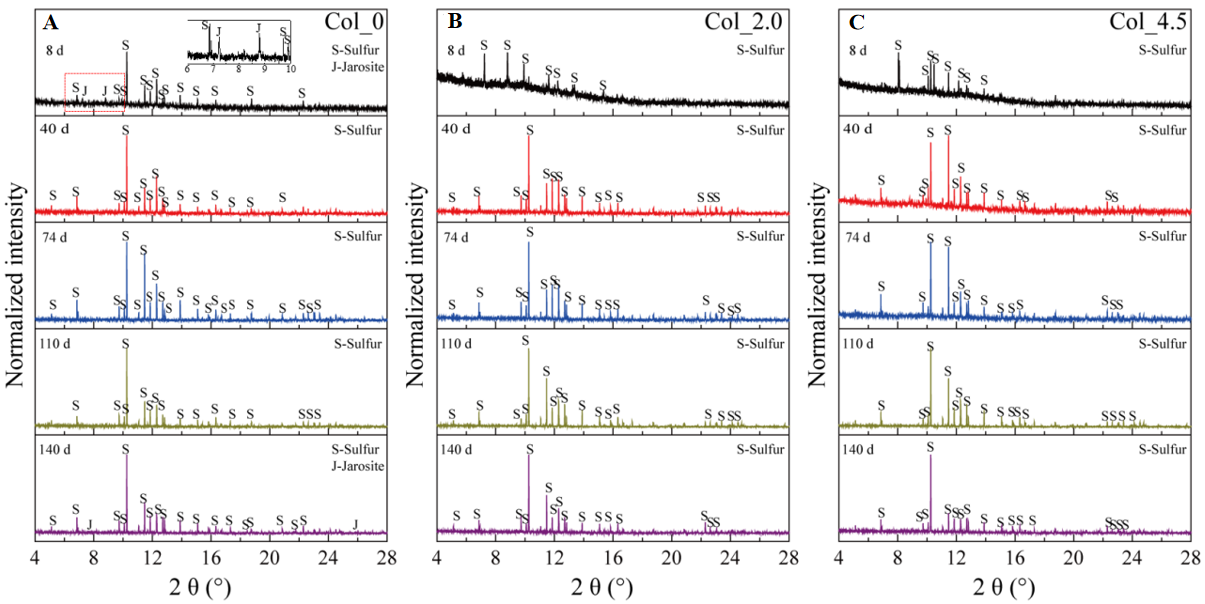


**Figure S5.** XRD patterns of bottom residues during the co-cultivation of *At. ferrooxidans* and *Aph. acidophilum* without (**A**) and with additions of 2.0 (**B**) and 4.5 (**C**) mM As(III). The internal image of the top of panel (**A**) represents the enlargement of the XRD patterns from 6 - 10 degree at day 8 for Col_0.


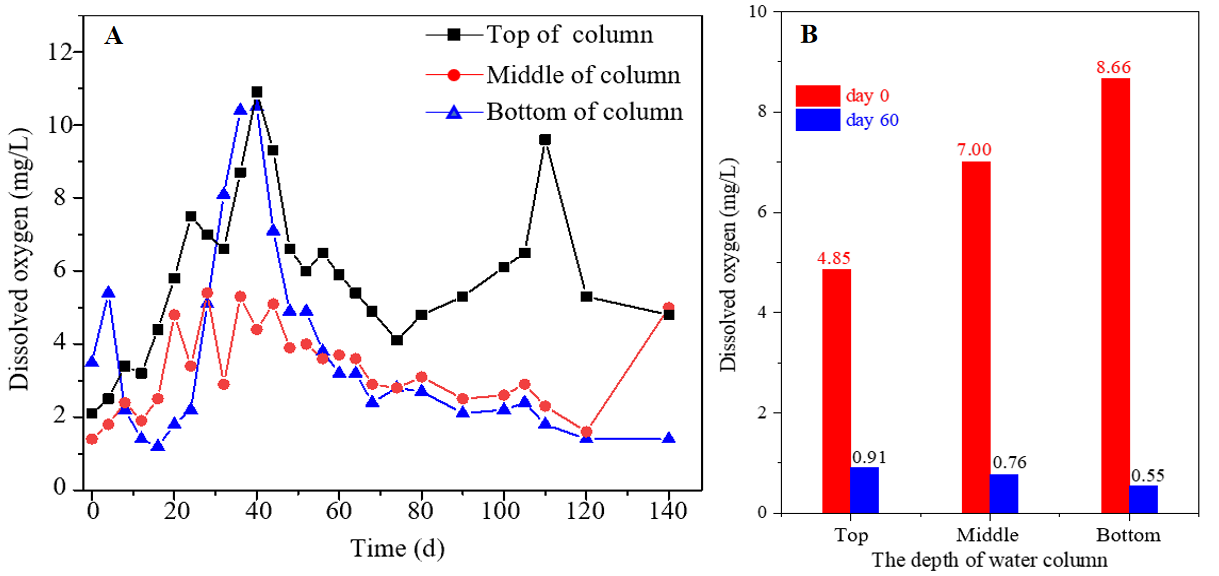


**Figure S6.** The change of dissolved oxygen at different depths of water columns (**A**); the dissolved oxygen for different depths (top, middle and bottom) of the sterile water column at days 0 and 60 (**B**). The DO of the water columns with *At. ferrooxidans* and *Aph. acidophilum* at different positions varied in a very wide range, and in general the DO at the bottom and the middle is lower than the top.


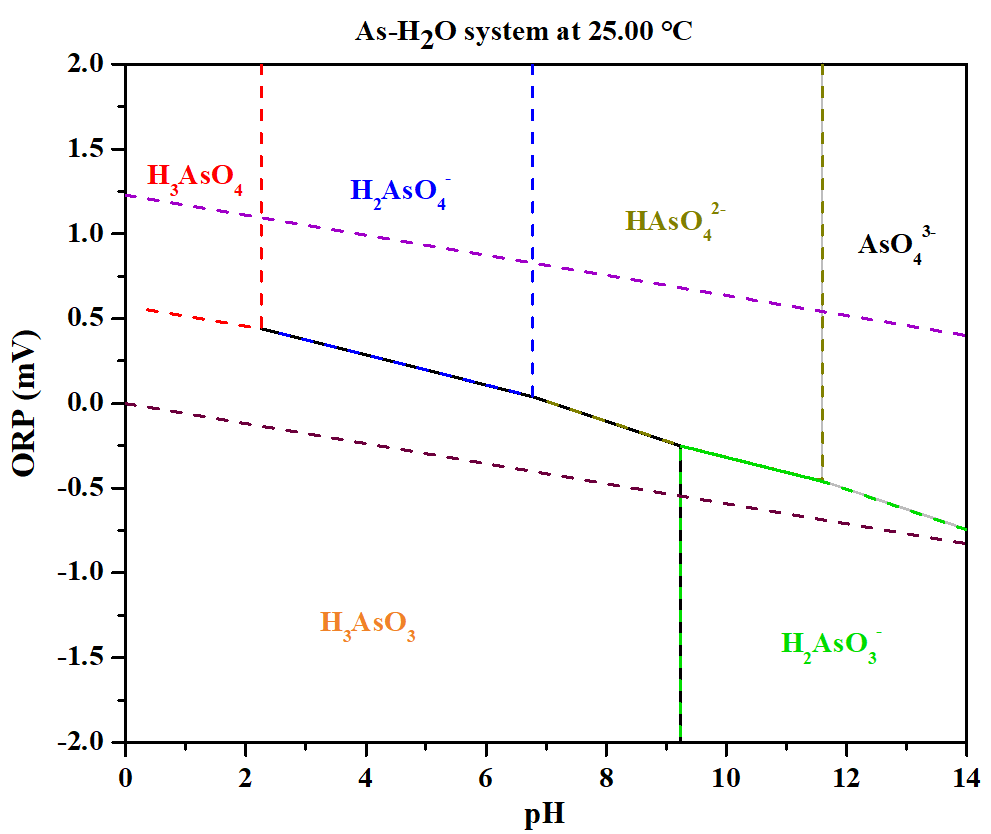


**Figure S7.** The pH-ORP diagram of As-containing phases in As-H_2_O system at 25 °C


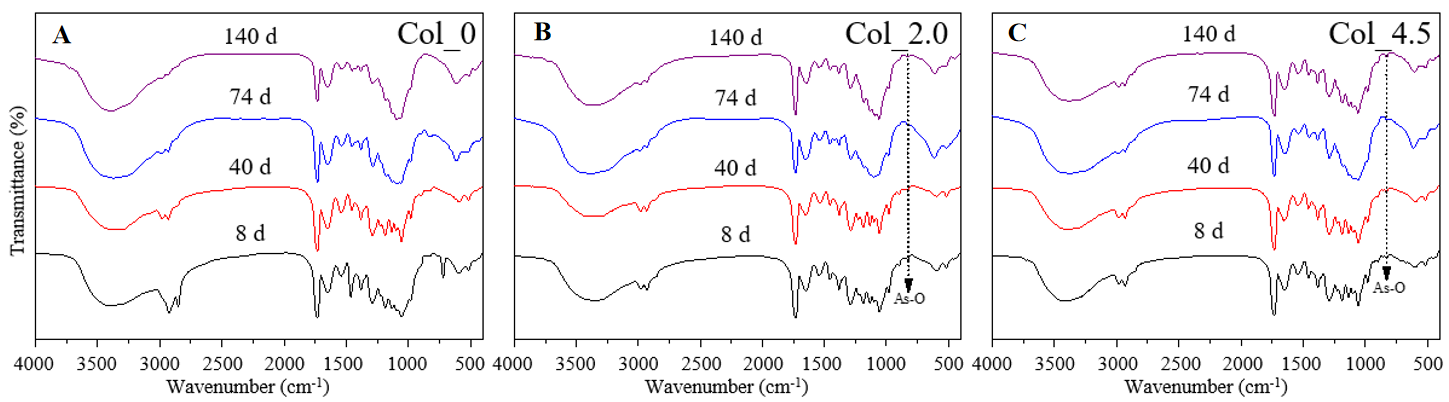


**Figure S8.** The FT-IR spectra of bacterial cells at the bottom of water columns during the co-cultivation of *At. ferrooxidans* and *Aph. acidophilum* without (**A**) and with additions of 2.0 (**B**) and 4.5 (**C**) mM As(III). The bacterial cells at the bottom were collected for FT-IR analyses to further verify whether As was adsorbed on the cell surface. By comparing with the case without As(III) (**A**), there occurred weak bands at 827 cm^-1^ and 829 cm^-1^ associating with As-O vibration in the spectra for the cases with As(III) added (**B** and **C**), indicating that that As(III) was adsorbed on the bacterial surface. This results also explain why the As concentrations at the bottom for Col_2.0 and Col_4.5 were lower than that at the other positions.


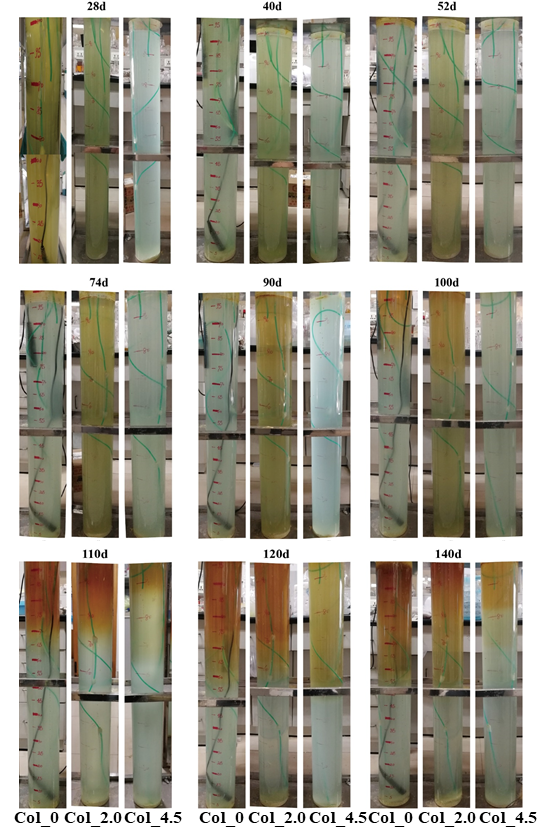


**Figure S9.** The images of Col_0, Col_2.0 and Col_4.5 at different time.

## Supplementary Tables

**Table S1.** The relative abundances of *At. ferrooxidans* and *Aph. acidophilum* for the top, middle and bottom of the water columns without (Col_0) and with additions of 2.0 (Col_2.0) and 4.5 (Col_4.5) mM As(III).

| **Time (d)** | The bacteria composition (*10^7^ cells/mL) and relative abundances (%) of *At. ferrooxidans* and *Aph. acidophilum* | | | | | |
| --- | --- | --- | --- | --- | --- | --- |
|  | Col_0 | | Col_2.0 | | Col_4.5 | |
|  | *At. ferrooxidans* | *Aph. acidophilum* | *At. ferrooxidans* | *Aph. acidophilum* | *At. ferrooxidans* | *Aph. acidophilum* |
| **Top** | | | | | | |
| 8 | 13.24 (97%) | 0.36 (3%) | 5.88 (75%) | 1.92 (25%) | 0.98 (13%) | 6.82 (87%) |
| 40 | 6.70 (94%) | 0.40 (6%) | 6.89 (93%) | 0.51 (7%) | 3.49 (62%) | 2.11 (38%) |
| 74 | 27.75 (96%) | 1.05 (6%) | 4.53 (46%) | 5.27 (54%) | 2.82 (47%) | 3.18 (53%) |
| 140 | 3.04 (51%) | 2.96 (49%) | 3.29 (47%) | 3.71 (53%) | 0.30 (10%) | 2.70 (90%) |
| **Middle** | | | | | | |
| 8 | 5.79 (93%) | 0.41 (7%) | 3.93 (82%) | 0.87 (18%) | 4.73 (91%) | 0.47 (9%) |
| 40 | 6.86 (88%) | 0.94 (22%) | 4.86 (76%) | 1.54 (24%) | 4.35 (95%) | 0.25 (5%) |
| 74 | 30.41 (97%) | 0.79 (3%) | 5.04 (49%) | 5.16 (51%) | 1.47 (37%) | 2.53 (63%) |
| 140 | 3.18 (24%) | 9.82 (76%) | 6.42 (92%) | 0.58 (8%) | 1.41 (35%) | 2.59 (65%) |
| **Bottom** | | | | | | |
| 8 | 14.33 (94%) | 0.87 (6%) | 10.33 (65%) | 5.47 (35%) | 3.20 (30%) | 7.60 (70%) |
| 40 | 29.30 (88%) | 3.90 (12%) | 55.05 (92%) | 4.95 (8%) | 6.26 (10%) | 54.54 (90%) |
| 74 | 40.59 (94%) | 2.61 (6%) | 36.92 (87%) | 5.28 (13%) | 17.82 (60%) | 11.98 (40%) |
| 140 | 5.20 (21%) | 19.80 (79%) | 22.67 (67%) | 11.33 (33%) | 15.06 (68%) | 6.94 (32%) |

**Table S2.** Peak parameters for As 3d spectra and percentages of different surface As components in total As on surface suspended matter for Col_2.0 and Col_4.5.

| As 3d_(5/2)_ | As(-I)-S | As(0) | As(I)-O | As(III)-O | As(V)-O | FeAsO_4_·2H_2_O-like phase |
| --- | --- | --- | --- | --- | --- | --- |
| BE (eV) | 40.9 | 41.8± 0.2 | 43.2± 0.3 | 44.1± 0.3 | 45.1± 0.2 | 45.6± 0.1 |
| Sample | (at. %) | (at. %) | (at. %) | (at. %) | (at. %) | (at. %) |
| Col_2.0-105 d | 7.89 | 6.17 | 12.07 | 29.95 | 25.77 | 18.15 |
| Col_4.5-120 d | 0 | 9.66 | 32.17 | 33.83 | 19.51 | 4.83 |

**Table S3.** Peak parameters for Fe 2p spectra and percentages of different surface Fe^2+^ and Fe^3+^ in total Fe on surface suspended matter for Col_0, Col_2.0 and Col_4.5.

| Fe 2p_(3/2)_ | Fe^3+^ | Fe^2+^ |
| --- | --- | --- |
| BE (eV) | 714.7± 0.1 | 711.9± 0.1 |
| Sample | (at. %) | (at. %) |
| Col_0-105 d | 40.39 | 59.61 |
| Col_2.0-105 d | 27.51 | 72.49 |
| Col_4.5-120 d | 24.76 | 75.24 |

**Table S4.** Fitted results of Fe L-edge XANES spectra of bottom residues during the cocultivation of *At. ferrooxidans* and *Aph. acidophilum* without and with As(III) added.

| Time (day) | Contribution of Fe(II) for total Fe (%) | | |
| --- | --- | --- | --- |
|  | Col_0 | Col_2.0 | Col_4.5 |
| 8 | 18.23 | 29.36 | 54.48 |
| 40 | 37.20 | 40.09 | 50.11 |
| 74 | 77.62 | 39.75 | 53.49 |
| 100 | 50.86 | 26.35 | 43.75 |
| 140 | 14.70 | 15.60 | 10.71 |

**Table S5.** Fitted results of S K-edge XANES spectra of bottom residues during the cocultivation of *At. ferrooxidans* and *Aph. acidophilum* without and with As(III) added.

| Sample | Time (day) | Percentage of contribution of reference spectra (%) | | |
| --- | --- | --- | --- | --- |
|  |  | S^0^ | Na_2_S_2_O_3_ | SO_4_^2-^ |
| Col_0 | 8 | 9.7 | - | 90.3 |
|  | 40 | 9.4 | - | 90.6 |
|  | 74 | 80.1 | 5.6 | 14.3 |
|  | 100 | 90.7 | 8.6 | 0.6 |
|  | 140 | 90.3 | 7.8 | 1.9 |
| Col_2.0 | 8 | - | - | 100 |
|  | 40 | 25.6 | - | 74.4 |
|  | 74 | 67.0 | - | 33.0 |
|  | 100 | 29.8 | - | 70.2 |
|  | 140 | 33.9 | 19.0 | 47.1 |
| Col_4.5 | 8 | - | - | 100 |
|  | 40 | 4.3 | - | 95.7 |
|  | 74 | 35.3 | - | 64.7 |
|  | 100 | 93.5 | 3.3 | 3.2 |
|  | 140 | 24.1 | 17.2 | 58.7 |

**Table S6.** Peak parameters for S 2p spectra and percentages of different surface sulfur components in total sulfur of bottom sediments for Col_0, Col_2.0 and Col_4.5.

| S 2p_(3/2)_ | | S^2-^ | S_2_^2-^ | S_n_^2-^ | S^0^ | SO_3_^2-^ | SO_4_^2-^ |
| --- | --- | --- | --- | --- | --- | --- | --- |
| BE (eV) | | 162.1 | 163.1 | 163.8 | 164.6 | 166.5 | 168.8 |
| Sample /day | | (at. %) | (at. %) | (at. %) | (at. %) | (at. %) | (at. %) |
| Col_0 | 8 | 0 | 8.04 | 6.53 | 2.84 | 2.13 | 80.47 |
|  | 40 | 0 | 12.82 | 25.21 | 4.01 | 2.19 | 55.77 |
|  | 74 | 0 | 4.82 | 87.82 | 1.07 | 0 | 6.29 |
|  | 110 | 0 | 4.39 | 90.21 | 1.69 | 0 | 3.72 |
|  | 140 | 0 | 4.01 | 90.98 | 2.01 | 0 | 3.00 |
| Col_2.0 | 8 | 0 | 0 | 0 | 0 | 0 | 100 |
|  | 40 | 7.61 | 43.28 | 6.30 | 1.08 | 1.88 | 39.84 |
|  | 74 | 4.32 | 17.79 | 2.88 | 4.32 | 1.64 | 69.04 |
|  | 110 | 12.39 | 28.27 | 27.93 | 2.36 | 1.14 | 27.92 |
|  | 140 | 7.62 | 16.71 | 16.71 | 4.83 | 2.23 | 51.01 |
| Col_4.5 | 8 | 0 | 0 | 0 | 0 | 0 | 100 |
|  | 40 | 4.83 | 6.92 | 1.22 | 1.22 | 0 | 85.81 |
|  | 74 | 3.13 | 79.98 | 4.47 | 0.90 | 0 | 11.52 |
|  | 110 | 5.04 | 75.43 | 5.04 | 1.46 | 0 | 13.39 |
|  | 140 | 12.77 | 26.17 | 14.25 | 5.10 | 2.55 | 39.15 |

**Table S7.** Peak parameters for As 3d spectra and percentages of different surface As components in total As of bottom sediments for Col_2.0 and Col_4.5.

| As 3d_(5/2)_ | | As(-I)-S | As(0) | As(I)-O | As(III)-O | As(V)-O | FeAsO_4_·2H_2_O-like phase |
| --- | --- | --- | --- | --- | --- | --- | --- |
| BE (eV) | | 40.9± 0.1 | 41.8± 0.2 | 43.2± 0.3 | 44.1± 0.3 | 45.1± 0.2 | 45.6± 0.1 |
| Sample /day | | (at. %) | (at. %) | (at. %) | (at. %) | (at. %) | (at. %) |
| Col_2.0 | 8 | 0 | 1.53 | 6.14 | 50.19 | 41.12 | 0 |
|  | 40 | 0 | 6.33 | 7.27 | 36.37 | 22.79 | 27.26 |
|  | 74 | 0 | 3.10 | 5.13 | 11.14 | 49.69 | 30.94 |
|  | 110 | 0 | 2.86 | 5.72 | 19.06 | 24.88 | 47.48 |
|  | 140 | 2.98 | 1.61 | 5.96 | 11.92 | 28.46 | 49.06 |
| Col_4.5 | 8 | 0 | 3.36 | 9.42 | 54.80 | 32.43 | 0 |
|  | 40 | 0 | 2.82 | 7.89 | 44.53 | 44.76 | 0 |
|  | 74 | 1.35 | 9.70 | 64.45 | 16.40 | 5.40 | 2.70 |
|  | 110 | 3.39 | 3.18 | 25.42 | 28.21 | 30.49 | 9.32 |
|  | 140 | 0 | 4.84 | 6.45 | 14.51 | 24.20 | 50.00 |
